# Efficacy of tubing technique with biomaterials compared to direct coaptation technique after peripheral neurotmesis in nerve healing and return to functionality in young adult rats: a systematic review protocol

**DOI:** 10.1186/s13643-020-01388-5

**Published:** 2020-05-28

**Authors:** Ana Camila Nobre de Lacerda Brito, Sara Emanuely Veríssimo Santos, Wilayane Alves Martins, Paulo César da Silva Queiroz, Wenddy Wyllie Damascena Sougey, Paula Ketilly Nascimento Alves, Kalline Lourenço Ribeiro, Maria Danielly Lima de Oliveira, Sílvia Regina Arruda de Moraes

**Affiliations:** 1 Neuropsychiatry and Behavioral Science Program, Federal University of Pernambuco, Avenida Professor Moraes Rego, 1235, Recife, 50670-901 Pernambuco Brazil; 2 Department of Anatomy, Neuromuscular Plasticity Laboratory, Federal University of Pernambuco, Recife, Pernambuco Brazil; 3 Department of Physiotherapy, Federal University of Pernambuco, Recife, Pernambuco Brazil; 4 Cellular and Tissue Biology Program, University of Sao Paulo, São Paulo, Brazil; 5 Therapeutic Innovation Program, Federal University of Pernambuco, Recife, Pernambuco Brazil; 6 Department of Biochemistry, Federal University of Pernambuco, Recife, Pernambuco Brazil

**Keywords:** Peripheral nerve injuries, Peripheral nerve, Neurosurgical procedure, Rat, Non-human

## Abstract

**Background:**

Peripheral nerves are constant targets of traumatic injury which may result in neurotmesis and which invariably requires surgical treatment. In view of this, tissue engineering studies developed biomaterials which were first tested in animal models and used as a guide for nerve stumps in the procedure in order to speed up the healing process. Therefore, the aim of this study is to evaluate the efficacy of biomaterials used in tubing technique on healing and histological and functional recovery after peripheral nerve neurotmesis in rats.

**Methods:**

We will search PubMed/MEDLINE, Embase, Web of Science, LILACS, and CENTRAL (from inception onwards). Grey literature will be identified through searching dissertation databases, guidelines, policy documents, and reports. We will include randomized and non-randomized trials conducted in young adult rats with peripheral neurometsis undergoing surgical repair through tubing technique with biomaterials. Primary outcomes will be histomorphometry, immunohistochemistry of the nerve tissue, and sciatic functional index. Secondary outcome will be nerve macroscopic evaluation. Two reviewers will independently screen all citations, full-text articles, and abstract data. Potential conflicts will be resolved through discussion. The methodological quality (or risk of bias) of individual studies will be appraised using an appropriate tool. If feasible, we will conduct random effects meta-analysis.

**Discussion:**

This systematic review of animal studies will identify, evaluate, and synthetize the evidence on the the efficacy of tubing technique with biomaterials compared to direct coaptation technique after peripheral neurotmesis in nerve healing and return to functionality.

**Systematic review registration:**

PROSPERO CRD42018106042.

## Background

Peripheral nerves are constant targets of traumatic injuries, resulting in interruption of nerve impulse transmission and loss or decreased sensation and muscle function in the innervated area. This type of injury affects individuals of all ages and can result in disability, representing a serious economic and social problem [1–4]. After a full or partial nerve injury, there is an important sequence of events as immediate production of potential shots of action, neuropeptide secretion and pro-inflammatory mediators at the injury site and central nervous system, activation of nociceptors and sensitization of silent nociceptors through release of inflammatory products or the expression of new receptors or ion channels, gene expression changes and the expression of various peptides and receptors, abnormal budding peripheral and central fibers, and receptive field changes and sensory modalities of damaged and intact peripheral fibers [5, 6].

It is known that the worst scenario for these injuries is one in which complete nerve separation occurs due to total disruption of axons, the myelin, and connective tissue components, with the latter being of great importance for promoting spontaneous recovery. Therefore, neurotmesis invariably requires surgical treatment in order to realign the nerve stumps and accelerate the nerve regeneration process [4, 7].

In the literature, the main types of surgical repair include direct nerve edge coaptation, which can lead to excessive strain, repair with interposition, where nerve autografting can be mentioned, and being associated with donor site morbidity, but can also be an allograft from processed cadaveric nerve or via pipes, which can be a vein graft or artificial tubing [4].

As each type of surgical repair has limitations, artificial tubes have been the main choice, mostly due to the limited availability of donor tissue for the performance of other methods [8, 9].

Therefore, studies on tissue engineering have been increasingly developed in order to develop tubes with biodegradable materials which are biocompatible, have low cytotoxicity, suppleness, and a suitable decomposition time which can be used as a support in order to accelerate the process of peripheral nervous tissue healing [10–12]. Although Konofaos and Halen [8] carried out a description of materials used to guide the nerve stumps, to date, there is no systematic review to provide data on the methodological quality of existing experimental studies which investigated the use of biomaterials in prepared tubes as adjuncts to surgically repair peripheral neurotmesis in an animal model.

## Objectives

The aim of this review is to evaluate the efficacy of tubing technique using biomaterials in the healing process based on rigorous methodology, and the histological and functional recovery after peripheral neurotmesis in young adult rats.

## Methods/design

### Study question

Do the surgical repair results of peripheral neurotmesis in experimental studies using tubing technique with biomaterials exhibit better healing process and functional recovery compared to those in which tubing was not used?

### Protocol and registration

This protocol was developed by a research team formed by members of Neuromuscular Plasticity Laboratory (A.C.N.L.B., W.A.M., P.C.S.Q., S.E.V.S., S.R.A.M.), members of the Biodevices Nanostructures Laboratory (Bionano) (K.L.R., M.D.L.O.), and members of the Program in Cellular and Tissue Biology (University of Sao Paulo) (W.W.D.S., P.K.N.A.). The present protocol has been registered within the PROSPERO database (registration number CRD42018106042) and is being reported in accordance with the reporting guidance provided in the Preferred Reporting Items for Systematic Reviews and Meta-Analyses Protocols (PRISMA-P) statement [13, 14] (see checklist in Additional file 1).

### Eligibility criteria

Studies will be selected according to the following criteria: population, interventions, comparisons, outcomes, and study design(s).

- Population: young adult rats (60–100 days or 250 g–500 g) which suffered peripheral neurotmesis induced by any method. Due to hormonal factors that may interfere with the neurotmesis healing, studies with rats with morbidities will be excluded
- Intervention: immediate surgical nerve repair with tubing technique with any biological or artificial biomaterial

The biomaterials used in the tubing technique are tubes that can be prepared from natural or synthetic materials and must be biocompatible, biodegradable, low cytotoxic, and have sufficient porosity to allow cell migration and vascularization. In addition, they are responsible for the mechanical protection of the tissue and are responsible for containing the substances responsible for regeneration within their lumen [15–17]. In order to only evaluate the biomaterial used, studies that accelerated nerve healing with drugs or stem cells will be excluded.

- Comparison: immediate surgical direct coaptation of nerve ends
- Outcome: the following primary outcomes were considered: histomorphometry (diameter of nerve fibers and axons, thickness of myelin sheath, amount of myelin fibers, and vasa nervorum) and immunohistochemistry (inflammatory markers) of the nerve tissue, sciatic functional index, and as a secondary outcome, the nerve macroscopic evaluation. This is a qualitative analysis where the alignment of the nerve stumps, biomaterial absorption, and quality of external and internal suture can be evaluated.
- Study designs: randomized controlled trials and non-randomised controlled trials will be included. We will exclude non-controlled studies, case reports, cross-over studies, literature reviews, letters to the editor, human studies, in vitro studies, studies with other animal models, or studies which did not perform the immediate surgical repair.

### Search strategy

First, we will search the following electronic databases: PubMed/MEDLINE, Embase, Web of Science, and LILACS via BVS (www.bvsalud.org) Cochrane Central Register of Controlled Trials (CENTRAL). A draft search strategy for PubMed/MEDLINE is provided in Additional file 2. The secondary source of potentially relevant material will be a search of the grey or difficult to locate literature, including two dissertation databases: ProQuest-Dissertations and Theses and Directory of Open Access Journals and Open Access Theses and Dissertations. We will perform hand-searching of the reference lists of included studies [18], relevant reviews, guidelines, relevant documents (e.g., official documents and government reports), and online sources from organizations such as the World Health Organization (WHO). No restrictions will be imposed with regard to language or publication date.

### Screening and selection methods

References will be managed in EndNote (version X7.0.1 New York City: Thomson Reuters, 2010), and duplicates will be removed. All articles identified from the literature search will be screened by two team members independently (ACNLB; SEVS). First, titles and abstracts of articles returned from initial searches will be screened based on the eligibility criteria outlined above. Second, full texts will be examined in detail and screened for eligibility. Third, references of all considered articles will be hand-searched to identify any relevant report missed in the search strategy. Any disagreements will be resolved by discussion to meet a consensus with a third reviewer (SRAM), if necessary. A flow chart showing details of studies included and excluded at each stage of the study selection process will be provided. Reasons for exclusion of potentially eligible studies will be recorded. IBM SPSS Statistics (version 20, IMB Corporation, 2011) will be used to evaluate the interobserver agreement, and the Kappa concordance index (*k* statistic ≥ 0.75) will be measured.

Full texts of potentially eligible studies will be obtained, and reviewers working independently will agree on the final included studies. The studies not meeting the inclusion criteria for the SR will be excluded. Duplicate articles will only be counted once.

### Data extraction

The data extracted from the selected articles will be organized on a chart specifically designed for the systematic review, which will contain the following:

- General characteristics of included studies: authors, date of publication, country of origin, objective, sample size, and main outcomes
- Intervention characteristics: type of biomaterial used in neurotmesis repair surgery, follow-up period
- Group characteristics: experimental groups, size of the group, age, gender, and weight of the animals used in the study

This relevant data will preferentially extracted from result tables in the selected manuscripts as mean ± standard deviation or median and interquartile range. If only individual data is present in the manuscript, the mean and standard deviation will be calculated from these values. If the data is not listed in the table, the text in the results topic will be carefully read for important information. If data is available in graphs only, the data is extracted manually using the Image J® (version 1.52a, Wayne Rasband, National Institutes of Health, USA) software. If data are not available in the text but mentioned in the manuscript, the authors will be contacted by email (up to 2 attempts).

### Risk of bias in individual studies

The bias risk of each included study will be classified through the items of the bias risk assessment tool and the methodological quality of the animal model research, Systematic Review Center for Laboratory Animals (SYRCLE) (Additional file 3). For each domain, the term “low” will be used to indicate low risk of bias, “high” to indicate high risk of bias, and “unclear risk of bias” when there is no clarity in the item identified in the article. This analysis will be carried out independently by two reviewers of this study. Disagreement between the review authors will be solved by including a third reviewer.

### Data synthesis

From the included studies, a narrative synthesis will be performed and, where possible, a meta-analysis will be presented. Studies will first be grouped according to the type of biomaterial used in nerve repair surgery. After studies have been combined into common groups, their characteristics such as sex of animals and nerve studied will be presented in summary tables. These groupings will be utilized to determine where categories of studies exist that has similar enough designs to allow for a meta-analysis of their reported outcomes.

If feasible, data points from primary studies will be used to perform random effects meta-analyses. Since heterogeneity is expected a priori, we will estimate the pooled mean differences or stardardised mean differences and its 95% confidence interval using the random effects model, where appropriate.

If a study has more than one treated group relevant for inclusion in the systematic review, the treated groups will be included as two separate studies, and the control group will be separated [19].

We will test the clinical heterogeneity by considering the variability in participant factors among trials (sex of animals and nerve studied) and trial factors (type of biomaterial used in nerve repair surgery). Statistical heterogeneity will be tested using Cochran’s *Q* with a *P* value of < 0.10 considered statistically significant and *I*^2^ statistic (0 to 40%: might not be important; 30 to 60%: may represent moderate heterogeneity; 50 to 90%: may represent substantial heterogeneity; 75 to 100%: considerable heterogeneity). We will try to explain the source of heterogeneity by subgroup analyses or sensitivity analyses [14, 20] considering as potential covariates: sex of animals (male vs female) and risk of bias in individual studies (e.g., low/moderate vs high risk).

Small study effects (or “publication bias” across studies) will be assessed by inspection of the funnel plots for asymmetry [19]and the Egger test [20], with the results considered to indicate potential small study effects when *P* values are < 0.10.

## Discussion

The present work provides a protocol for systematic review of experimental studies that have used the tubing technique with biomaterials in the healing process, histological, and functional recovery after peripheral neurotmesis in young rats.

The results of the systematic review from this study will be useful for scientific research in neurosurgery, since when evaluating the methodological quality of studies with this theme, it indicates which biomaterials used in animal models had results from an appropriate methodology, favoring a possible external validity for clinical studies.

Although in an animal model, it is not possible to observe homogeneity between the studies, even so, due to the great use of rats in experimental research to evaluate a new biomaterial to be used in the surgical repair of a nerve injury, this animal model was chosen as the inclusion criteria in the systematic review [21].

The chosen theme is due to the increase in the injuries of the peripheral nervous system, which culminate in the reduction of the quality of life and partial or total loss of the productive activities of the patients, and, consequently, increase the expenses of public health and social security [22, 23]. As well as due to the great availability of artificial conduits and the low level of methodological and comparative evidence of these materials, which leaves the surgeon in charge of choosing the conduit to be used in the surgery [24].

Thus, the systematic review will discuss the effectiveness of biomaterials used in the tubing technique in the healing process after neurotmesis in young adult rats.

## Supplementary information

**Additional file 1.** : PRISMA-P 2015 Checklist.

**Additional file 2.** : Table 1. Example search strategy for MEDLINE database.

**Additional file 3.** : Table 2. SYRCLE risk of bias (ROB) tool.

## Data Availability

Not applicable.

## Abbreviations

PICOS: Population Intervention, Comparator, Outcome, Study Designs
SYRCLE: Systematic Review Center for Laboratory Animal Experimentation
ARRIVE: Animal Research: Reporting of In Vivo Experiments
PROSPERO: International prospective register of systematic reviews
A.C.N.L.B.: Ana Camila Nobre de Lacerda Brito
W.A.M.: Wilayane Alves Martins
P.C.S.Q.: Paulo César da Silva Queiroz
S.E.V.S.: Sara Emanuely Veríssimo Santos
S.R.A.M.: Sílvia Regina Arruda de Moraes
K.L.R.: Kalline Lourenço Ribeiro
M.D.L.O.: Maria Danielly Lima de Oliveira
W.W.D.S.: Wenddy Wyllie Damascena Sougey
P.K.N.A.: Paula Ketilly Nascimento Alves
IL-1β: Interleucina1 beta
NGF: Nerve growth factor
iNOS: Inducible nitric oxide synthase
TNF-α: Tumor necrosis factor
VEGF: Vascular endothelial growth factor
