# Supplementary material for: Efficacy of tubing technique with biomaterials compared to direct coaptation technique after peripheral neurotmesis in nerve healing and return to functionality in young adult rats: a systematic review protocol
Source: Syst Rev. 2020 May 28;9:118. doi: 10.1186/s13643-020-01388-5 (PMC7254672; doi:10.1186/s13643-020-01388-5)
Supplement: Supplementary file 2 — Additional file 2. : Table 1. Example search strategy for MEDLINE database. [file 13643_2020_1388_MOESM2_ESM.docx]

**Efficacy of tubing technique with biomaterials compared to direct coaptation technique after peripheral neurotmesis in nerve healing and return to functionality in young adult rats: a systematic review protocol.**

**Table 1 -** Example search strategy for MEDLINE database.

| **Search** | **Search strategy** |
| --- | --- |
| #1 | “Peripheral Nervous System” [Mesh] |
| #2 | "Peripheral Nerves"[Mesh] |
| #3 | “Peripheral Nerve” |
| #4 | #1 OR #2 OR #3 |
| #5 | “Trauma, Nervous System” [Mesh] |
| #6 | "Peripheral Nerve Injuries"[Mesh] |
| #7 | “peripheral nerve injury” |
| #8 | “peripheral nerve damage” |
| #9 | “peripheral neurotmesis” |
| #10 | #5 OR #6 OR #7 OR #8 OR #9 |
| #11 | "Neurosurgical Procedures"[Mesh] |
| #12 | “Neurosurgical Procedure” |
| #13 | “Peripheral Nerve Repair” |
| #14 | “Peripheral Nerve Surgical” |
| #15 | “Peripheral Nerve Tubulization” |
| #16 | #11 OR #12 OR #13 OR #14 OR #15 |
| #17 | "Humans"[Mesh] |
| #18 | #4 AND #10 AND #16 NOT #17 |
