# Supplementary material for: Efficacy of tubing technique with biomaterials compared to direct coaptation technique after peripheral neurotmesis in nerve healing and return to functionality in young adult rats: a systematic review protocol
Source: Syst Rev. 2020 May 28;9:118. doi: 10.1186/s13643-020-01388-5 (PMC7254672; doi:10.1186/s13643-020-01388-5)
Supplement: Supplementary file 3 — Additional file 3. : Table 2. SYRCLE risk of bias (ROB) tool. [file 13643_2020_1388_MOESM3_ESM.docx]

| Item | Type of bias | Domain | Domain description | Review authors' judgement |
| --- | --- | --- | --- | --- |
| 1 | Selection bias | Sequence generation | Describe the methods used, if any, to generate the allocation sequence in sufficient detail to allow an assessment of Whether it should produce comparable groups. | Was the allocation sequence adequately generated and applied?^a^ |
| 2 | Selection bias | Baseline characteristics | Describe all the possible prognostic factors or animals characteristics, if any, that are compared in order to judge whether or not intervention and control groups were similar at the start of the experiment. | Were the groups similar at baseline or were they adjusted for confounders in the analysis? |
| 3 | Selection bias | Allocation concealment | Describe the method used to conceal the allocation sequence in sufficient detail to determine whether intervention allocations could have been foreseen before or during enrollment. | Was the allocation adequately concealed? ^a^ |
| 4 | Performance bias | Random Housing | Describe all measures used, if any, to house the animals randomly within the animal room. | Were the animals randomly housed during the experiment? |
| 5 | performance bias | Blinding | Describe all measures used, if any, to blind trial and caregiver researchers from knowing which animals received each intervention. Provide any information relating to whether the intended blinding was effective. | Were the caregivers and/or investigators blinded from knowledge of which animals received each intervention during the experiment? |
| 6 | Detection bias | Random outcome assessment | Describe whether or not animals were selected at random for outcome assessment, and which methods to select the animals, if any, were used. | Were animals selected at random for outcome assessment? |
| 7 | Detection bias | Blinding | Describe all measures used, if any, to blind outcome assessors from knowing which animals received each intervention. Provide any information relating to whether the intended blinding was effective. | Was the outcome assessor blinded? |
| 8 | Attrition bias | Incomplete outcome date | Describe the completeness of outcome data for each main outcome, including attrition and exclusions from the analysis. State whether attrition and exclusions were reported, the numbers in each intervention group (compared with the total randomized animals), reasons for attrition or exclusions, and any re-inclusions in analyzes for the review. | Were incomplete outcome data adequately addressed? ^a^ |
| 9 | Reporting bias | Selective outcome reporting | State how selective outcome reporting was examined and what was found. | Are reports of the study free of selective outcome reporting? ^a^ |
| 10 | Other | Other sources of bias | State any important concerns about bias not covered by other domains in the tool. | Was the study apparently free of other problems that could result in high risk of bias? ^a^ |
| ^a^ Items in agreement with the items in the Cochrane Risk of Bias tool. Hooijmans et al. BMC Medical Research Methodology 2014 14:43 doi: 10.1186 / 1471-2288-14-43. | | | | |

**Efficacy of tubing technique with biomaterials compared to direct coaptation technique after peripheral neurotmesis in nerve healing and return to functionality in young adult rats: a systematic review protocol.**

**Table 2 -** SYRCLE risk of bias (ROB) tool
